# The English Conjoint Course

**Published:** 1921-08-27

**Authors:** 


### THE ENGLISH CONJOINT COURSE.

The student who is desirous of taking the Conjoint qualification—that is to say, the double diploma of Member of the Royal College of Surgeons of England and Licentiate to the Royal College of Physicians of London—has to comply with the regulations laid down for medical qualifications generally. When he has passed a recognised preliminary examination he has to show that he has attended lectures and practical work in chemistry, physics, and biology before he is allowed to proceed to the first, second, or third part of his "first." The subjects for the first examination are chemistry, physics, and biology. In the first two parts the examination is partly written and partly practical; in the third part it is written and oral. The practical chemistry consists of simple quantitative analysis, and candidates usually find the test a comparatively easy one. Assuming that the student has had some preliminary training, such as can usually be obtained at the general schools from which he passes his preliminary, he ought not to take a longer time than six months to pass the three parts of the "first." The biology required for the first examination is elementary.

Having passed the first two parts of it, the student commences his study of anatomy and physiology. He must study these for twelve months during the ordinary session before he can enter for his second examination, and during that time he must have actually dissected the several "parts," and must have attended a course of instruction in histology and practical physiology, together with a course of lectures (six months) in physiology and anatomy at a recognised medical school. He must also attend a course of lectures on pharmacology, and receive recognised instruction in practical pharmacy.

The second examination is both oral and written, and consists of two parts: Part I., anatomy and physiology, and Part II., pharmacology and materia medica. Here, too, as in the "first," the questions are straightforward, and the student who has displayed average diligence in his studies should not have much difficulty in satisfying the examiners at the end of his second year. The *viva voces* are purely objective, and the candidate is rarely asked to describe any structure. Part II. consists of a *viva-voce* examination only.

For the final or qualifying examination the student must produce evidence of having attended at a recognised medical school specified courses of lectures on medicine, surgery, midwifery, pathology (including pathological histology and bacteriology) and therapeutics, forensic medicine and insanity, and public health; of having received systematic practical instructions in these subjects; and of having performed operations on the dead body. In addition he must show that he has attended, since passing his second examination, the practice of medicine and surgery, demonstrations in the *post-mortem* room, clinical lectures on medicine and surgery and diseases of women, and of having served as clerk and dresser in the wards. He must also have obtained a certificate of instruction and proficiency in the administration of anæsthetics, and have attended special classes in ophthalmology, in fevers, in lunacy, and in vaccination, and must have conducted twenty labours. In addition he must be twenty-one years of age or older.

The examination is in three parts, medicine, surgery, and midwifery, and the student is allowed to take the examination at the completion of five years of medical study, provided twenty-four months of professional study have been completed since passing the second examination. The examination comprises four papers of six questions, and a *viva voce*. In surgery and medicine there are additional *viva voces* in anatomy and pathology, together with a long *viva* on "clinical cases."

The Royal Colleges also grant special Diplomas in Public Health, Tropical Medicine and Hygiene, Ophthalmic Medicine and Surgery, and in Psychological Medicine, particulars of which can be obtained from the Secretary of the Conjoint Board, Examination Hall, 8-11 Queen Square, Bloomsbury, London, W.C. 1.

